# *Hafnia alvei* infection complicated by chorioamnionitis and septicemia: a case report and review of literature

**DOI:** 10.3389/fmed.2026.1840743

**Published:** 2026-06-08

**Authors:** Hong Wang, Hong Li, Haiyan Wang, Jin Zhang

**Affiliations:** 1 Department of Anesthesiology, Shijiazhuang Obstetrics and Gynecology Hospital, Shijiazhuang, Hebei, China; 2 Department of Anesthesiology, The Second Hospital of Hebei Medical University, Shijiazhuang, China

**Keywords:** chorioamnionitis, gestational diabetes mellitus, *Hafnia alvei*, multidisciplinary management, septicemia

## Abstract

**Background:**

*Hafnia alvei* (*H. alvei*) is an opportunistic pathogen that rarely causes extraintestinal infections. Chorioamnionitis involving this bacterium, though uncommon, can be life-threatening and rapidly progress to maternal septic shock. However, in the setting of polymicrobial infection—particularly when co-existing with *Escherichia coli*, a well-recognized pathogen in obstetric sepsis—the severe clinical manifestations cannot be attributed solely to *H. alvei*.

**Case summary:**

We report a case of a 27-year-old primipara at 33 + 5 weeks of gestation presenting with low-grade fever, lower abdominal pain, and tachycardia. Given high suspicion of intrauterine infection, a multidisciplinary consultation was conducted prior to delivery. The obstetric team diagnosed chorioamnionitis with risks of maternal septic shock and fetal distress. An emergency cesarean section was performed, during which placental surface swab and aerobic and anaerobic blood cultures were collected, followed by initiation of broad-spectrum antimicrobial therapy. Postoperatively, the patient’s condition deteriorated, requiring intensive care and norepinephrine administration to maintain hemodynamic stability. Empirical treatment with meropenem was administered to control infection. The preterm infants suffered from severe infections and survived after 3 weeks of treatment. Blood cultures obtained postoperatively revealed a rare mixed infection of *H. alvei* and *E. coli*.

**Conclusion:**

This case suggests that although *H. alvei* is relatively rare in extraintestinal infections, it may become an important pathogen in obstetrics. The rapid progression of the patient’s condition and the lack of definitive treatment guidelines underscore the importance of early clinical recognition and the use of appropriate empirical antibiotic therapy based on local antimicrobial susceptibility patterns. This report supplements the literature on *H. alvei* infection during pregnancy and confirms the critical role of blood cultures in guiding targeted treatment.

## 1 Introduction

*Hafnia alvei* is a Gram-negative, facultatively anaerobic bacterium belonging to the Enterobacteriaceae family. Although it is primarily recognized as a commensal organism inhabiting the gastrointestinal tracts of humans and animals, *H. alvei* has been implicated in rare cases of extra-intestinal infections (1, 2). Existing literature predominantly documents its role as an opportunistic pathogen in sporadic instances of bacteremia, pneumonia, and urinary tract infections, particularly among immunocompromised hosts (3–5). However, its pathogenic potential in immunocompetent individuals, especially within obstetric contexts, remains undercharacterized, with only isolated case reports available.

Chorioamnionitis is a leading cause of morbidity and mortality in pregnant women and neonates, primarily caused by ascending infections from Group B Streptococcus, *Escherichia coli*, and Ureaplasma species (6–8). *H. alvei* infection exhibits unique clinical characteristics: its rarity often leads to delayed diagnosis and may rapidly progress to maternal septic shock and multiple organ dysfunction syndrome (9, 10). The nonspecific clinical manifestations further complicate early diagnosis. Currently, there is no standardized treatment protocol for *H. alvei* infection during pregnancy, and the scarcity of cases limits the development of evidence-based therapeutic recommendations.

This report presents a rare case of polymicrobial infection in a primipara, caused by *H. alvei* and *E. coli*, leading to chorioamnionitis complicated by septic shock. The case manifested as rapid hemodynamic failure and paralytic ileus. Given that *E. coli* is a well-established pathogen capable of causing severe chorioamnionitis and maternal septic shock, the presence of this organism alone is sufficient to explain the clinical severity. Therefore, while *H. alvei* represents an unusual microbiological finding, its distinct pathogenic contribution in this case remains uncertain. Nevertheless, this case highlights the importance of comprehensive microbiological diagnosis in polymicrobial obstetric sepsis and the need for prompt, broad-spectrum antimicrobial therapy.

## 2 Case presentation

A 27-year-old primigravida at 33 weeks and 5 days of gestation was admitted to our institution with an 8-h history of progressive lower abdominal pain, pyrexia, and maternal tachycardia.

The current pregnancy was achieved via embryo transfer in our hospital’s reproductive medicine center due to fallopian tube obstruction. After she became pregnant, she had no sexual intercourse. At 16 weeks of gestation, mild vaginal bleeding occurred, and she was hospitalized for tocolysis with intravenous magnesium sulfate and oral dydrogesterone 10 mg three times daily.

She was complicated by GDM characterized by a hemoglobin A1c level of 6.2%. With dietary management alone, fasting blood glucose ranged from 4.5 to 6.4 mmol/L, and 2-h postprandial glucose ranged from 6.8 to 10.5 mmol/L.

**Figure 1:**
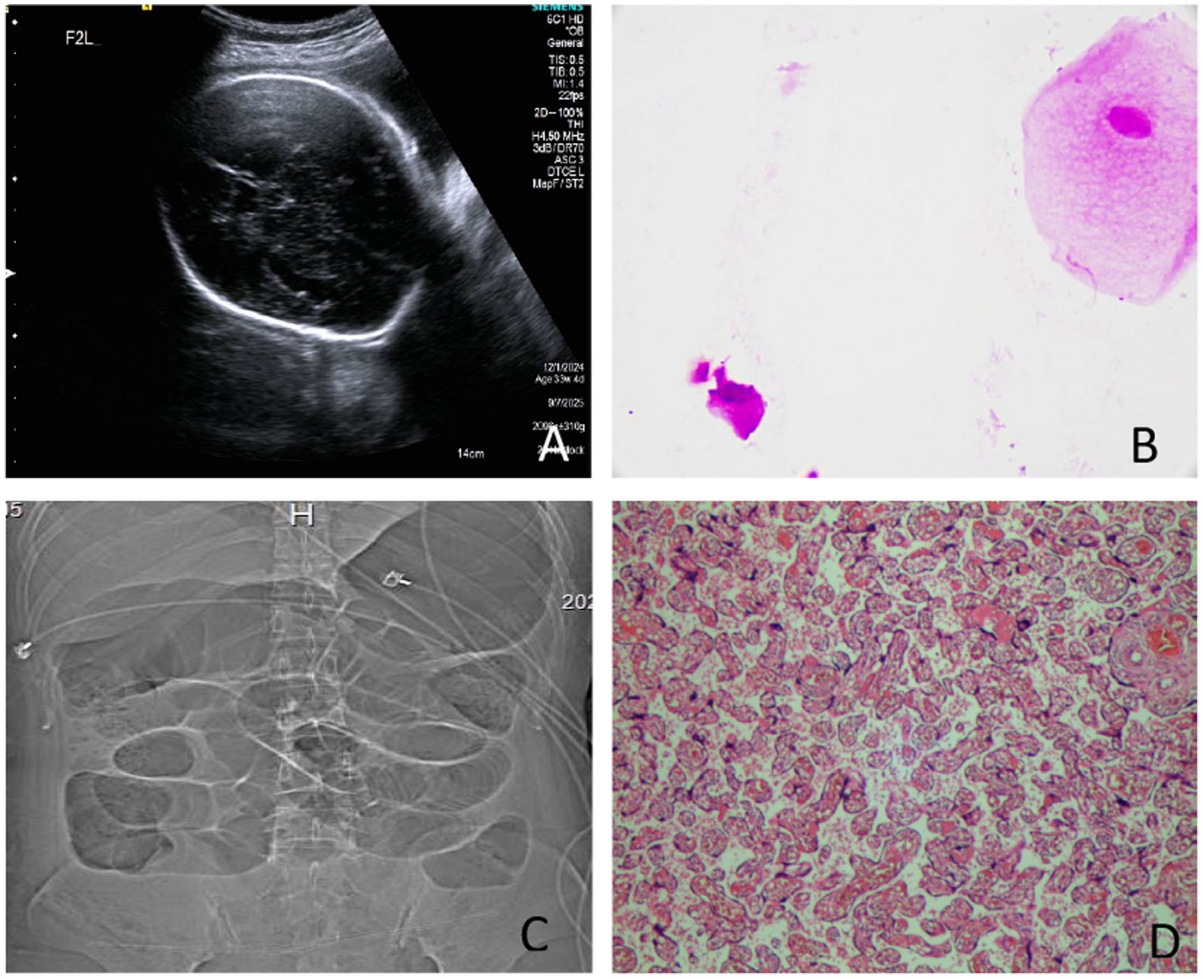
Clinical, radiological, and pathological findings. **(A)** Fetal ultrasound revealed estimated gestational age approximately 33^+1^ weeks for fetus F1 (right) and 32^+5^ weeks for fetus F2 (left), with estimated fetal weights of 2096 ± 310 g and 2080 ± 308 g, respectively. Placental maturity grade was I for both fetuses. Amniotic fluid pocket depths were 5.4 cm and 4.1 cm, respectively. **(B)** Vaginal microbiota analysis showed that the bacterial flora was inhibited, suggesting disruption of normal vaginal microbiota. **(C)** Radiographic evaluation of the abdominal plate was highly suggestive of intestinal obstruction, consistent with paralytic ileus. **(D)** Histopathological examination of placental tissue confirmed the presence of acute chorioamnionitis, with neutrophil infiltration of the chorionic plate and umbilical cord. PROM, premature rupture of membranes; IV, intravenous; IM, intramuscular; q12h, every 12 h; q8h, every 8 h; ICU, intensive care unit; NICU, neonatal intensive care unit.

Notably, she was hospitalized at 32 weeks of gestation due to threatened preterm labor. A detailed management plan was implemented as follows: dietary guidance was provided, and the dose of insulin aspart was adjusted accordingly. Fasting blood glucose was controlled at 3.5–4.8 mmol/L, and 2-h postprandial blood glucose was controlled at 6.1–7.5 mmol/L. Tocolysis was administered with ritodrine, and fetal lung maturation was promoted with dexamethasone at a dose of 6 mg intramuscularly every 12 h for a total of four doses. During this admission, ultrasound revealed a viable twin intrauterine pregnancy (Figure 1A).

Vaginal microbiota test results revealed pH 4.60 (reference range: 3.8–4.5), no dominant bacterial flora, and a positive hydrogen peroxide test (Figure 1B). A routine high vaginal swab culture identified *H. alvei* as the microbial isolate. Antimicrobial susceptibility testing demonstrated that the *H. alvei* isolate was susceptible to piperacillin-tazobactam, carbapenems, cephalosporins, and fluoroquinolones. Given the absence of clinical infection symptoms and the resolution of uterine contractions following tocolytic therapy, no targeted antibiotic treatment for *H. alvei* was initiated. After 12 days of hospitalization, the patient was discharged.

Upon admission at 33 weeks and 5 days of gestation, vital signs were unstable with a body temperature of 37.8 °C and a heart rate of 114 beats/min. Palpable tenderness was detected in the lower abdominal region. Sterile speculum examination confirmed the diagnosis of premature rupture of membranes.

Laboratory investigations revealed leukocytosis (18.2 × 10^9^/L) with a predominance of neutrophils (85%). with elevated C-reactive protein (25.2 mg/L) and procalcitonin (0.32 ng/mL). Elevated fasting blood glucose levels (6.28 mmol/L) and hypoalbuminemia (serum albumin: 25.4 g/L) were observed.

Given the continuous deterioration of the parturient’s condition, we promptly organized a multidisciplinary consultation including internal medicine, anesthesiology, neonatology, and obstetrics and decided to perform an emergency cesarean section. A diagnosis of sepsis was highly suspected; therefore, dexamethasone for fetal lung maturation was not administered.

The following management plan was formulated: preoperative preparation: Blood cultures were obtained prior to administer intravenous piperacillin-natamoxazole sodium. If the infection worsens and sepsis occurs, meropenem should be directly administered for anti-infective treatment. Initiate intravenous fluid resuscitation and maintain maternal systolic blood pressure above 100 mmHg to ensure adequate placental perfusion. Anesthetic management: Perform spinal-epidural combined anesthesia based on maternal hemodynamic stability and urgency, with close monitoring of vital signs and prompt management of hypotension using vasopressors.

During the procedure, we observed first-degree meconium contamination of the amniotic fluid, uterine tissue edema, and significant uterine atony. Immediate intraoperative sampling was performed for placental surface swab and blood cultures.

The patient’s condition rapidly deteriorated postoperatively, presenting with characteristics of high fever (40 °C), tachypnea (28 breaths/min), and hypotension (85/58 mmHg). Physical examination revealed significant abdominal distension accompanied by recurrent vomiting. Laboratory tests revealed a markedly elevated white blood cell count (31.7 × 10^9/L), an extremely high procalcitonin level (56.86 ng/mL). It also revealed increased serum creatinine (100 μmol/L), and hypofibrinogenemia (1.52 g/L). Abdominal CT demonstrated diffuse dilatation of small intestinal loops with multiple air-fluid levels (Figure 1C), suggestive of severe paralytic ileus. The clinical and imaging findings collectively aligned with the pathologic features of multiple organ dysfunction syndrome. The comprehensive treatment included hemodynamic stabilization, meropenem administration for infection control, intravenous insulin therapy for glycemic regulation, and nutritional support.

On postoperative day 3, placental surface swab and blood cultures grew *H. alvei* and *E. coli*, with negative results for anaerobic bacteria, Listeria, *Ureaplasma urealyticum*, and Mycoplasma. Placental histopathological examination confirmed the diagnosis of severe acute chorioamnionitis with funisitis (Figure 1D). Histopathological images of the placenta at 10×, 20×, and 40 × magnification are shown in the Supplementary material.

After 5 days, the patient was transferred to the postpartum ward, where antibiotic therapy was adjusted to piperacillin-tazobactam (4.5 g every 8 h) based on antimicrobial susceptibility testing. The patient was discharged on postoperative day 17 following complete recovery. The patient had a good prognosis during the three-month follow-up.

**Figure 2:**
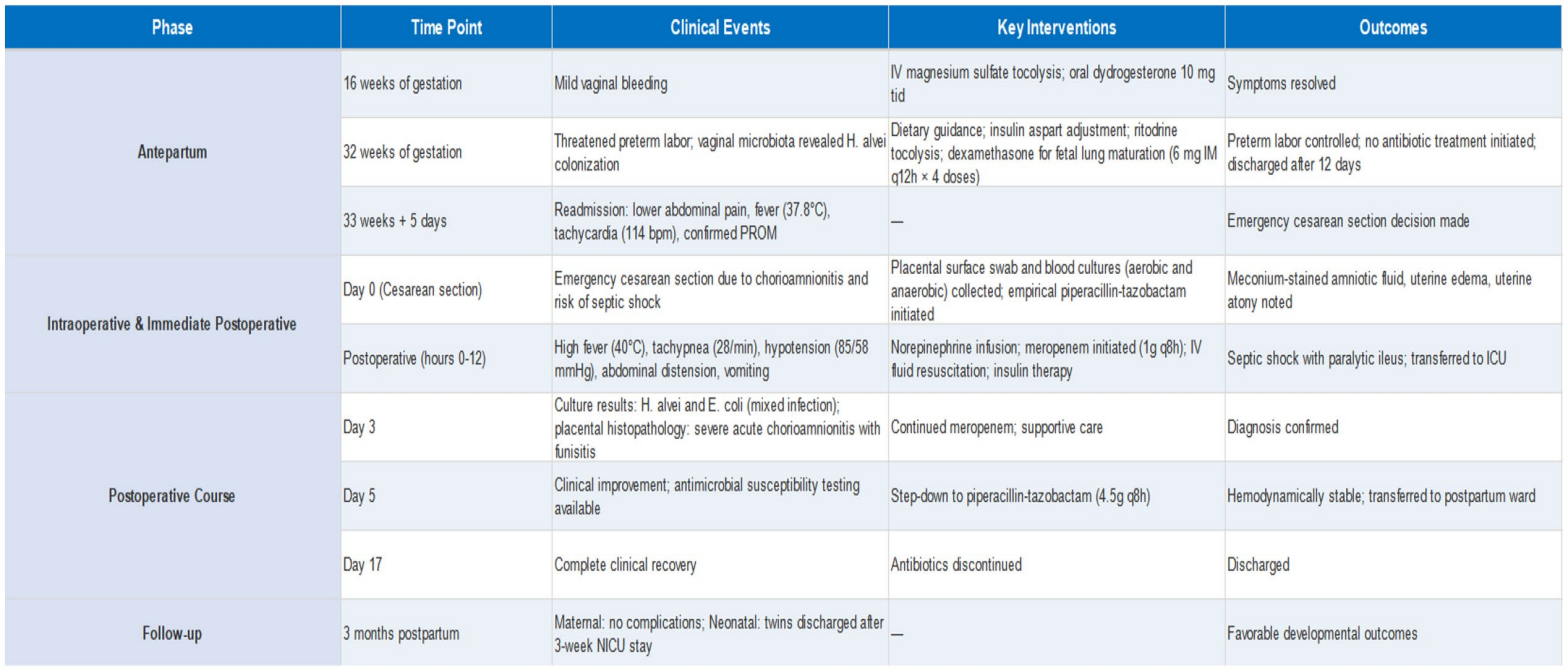
Clinical timeline of the episode of care. PROM, premature rupture of membranes; IV, intravenous; IM, intramuscular; q12h, every 12 h; q8h, every 8 h; ICU, intensive care unit; NICU, neonatal intensive care unit.

First twin (female, 2,260 g, Apgar 9/10/10) was delivered from grade I meconium-stained fluid, and second twin (female, 2070 g, Apgar 9/10/10) from clear fluid, both with normal umbilical cords. The twin had hematosepsis, acute respiratory distress syndrome, hyperbilirubinemia (early onset, phototherapy), and intracranial hemorrhage, managed with mechanical ventilation, vitamin K1. The twins were discharged after a 3-week hospitalization in the neonatal intensive care unit. Three-month follow-up demonstrated favorable developmental outcomes without complications (Figure 2).

### 2.1 Patient’s perspective

To assess patient perspectives, a structured interview was conducted within 72 h after emergency cesarean section. The interview evaluated the patient’s understanding, acceptance, and concerns regarding the importance of blood glucose management, the emergency procedure, peripartum antibiotic use, and the collection of placental swab and blood cultures. She reported good understanding and acceptance of all procedures. Documented concerns included postoperative pain, fear of breastfeeding interference, and potential antibiotic-related risks for the neonate. These findings highlight the need to incorporate patient-reported experiences into routine emergency obstetric care and future research.

## 3 Discussion

*H. alvei* is an opportunistic pathogen that primarily colonizes the intestinal microbiota, with occasional reports of causing respiratory and urinary tract infections (2). However, documented cases of obstetric infections caused by this bacterium remain extremely rare (Table 1).

**Table 1:** Literature review on extraintestinal infection of *H. alvei*.

Case	Age (years)	Medical history	Presentation	Diagnosis	Microbiology	Antibiotic treatment	Outcome
Ignaszewski 2016 (17)	39 F	Laparoscopic cholecystectomy for acute cholecystitis	Fever, chills, tachycardia, tachypnea, hypotension	Sepsis	*Enterococcus faecalis* and *H. alvei*	Piperacillin/tazobactam Levofloxacin and amoxicillin/clavulanate	Cure
Mufadda 2014 (13)	53 M	HIV, hepatitis C, hypertension, and diabetes	Left chest pain, cough with yellow sputum, and flu-like symptoms	Pneumonia	*H. alvei*	Broad-spectrum antibiotics (definition and duration unspecified)	Cure
Loulergue 2007 (14)	78 M	A pacemaker was implanted due to atrioventricular block	The intrinsic hypermetabolic state of the myocardium	Bacteremia, Endocarditis	*H. alvei*	Cephalosporins and Gentamicin 6 weeks total duration	Cure
Loya 2015 (18)	95 F	Stage 3b chronic kidney disease, hypertension, and Bonnet syndrome	Cough, high fever with chills, dyspnea	Pneumonia, pyothorax	*H. alvei*	Piperacillin/Tazobactam and Ciprofloxacin 4 weeks total duration	Cure
Herrera 2025 (15)	70 F	Cryptogenic cirrhosis after liver transplantation, advanced pancreatic cancer	Fever	Sepsis, urinary tract infection	Hafnia and Klebsiella acidophilia	Vancomycin and Ciprofloxacin	Dead
Redondo 2005 (19)	85 M	Moderate chronic obstructive pulmonary disease	Fever, cough, dyspneafever, cough, dyspnea	Pneumonia and bacteremia Pneumonia and bacteremia	*H. alvei*	NA	Dead
Candoni 2004 (16)	60 F	Diffuse large B-cell lymphoma	Fever, diarrhea, and abdominal pain	Abdominal abscess septicemia	*H. alvei*	Imipenem/cilastatin	Cure
Méndez 2021 (20)	48 M	NA	Fever, dyspnea, and hypoxemia	Pneumonia septic shock	SARS-CoV-2, *H. alvei*	Meropenem and Lincomycin	Cure
Palaniswamy 2009 (21)	75 M	Coronary artery disease	Right upper quadrant pain	Gangrenous Cholecystitis	*H. alvei*	Fluoroquinolones	Dead
Cutuli 2021 (22)	52 M	NO	Respiratory distress	Pneumonia	COVID-19, *H. alvei*	Meropenem	Cure
Crandall 2006 (23)	11 M	NA	Erythematous papules, abdominal pain, persistent vomiting, and decreased urine output	Hemolytic uremic syndrome	*H. alvei*	NA	Cure
Lim 2023 (24)	23 M	NA	Hemoptysis, mild dyspnea, pleuritic chest pain, fevers, and chills	Community-acquired cavitary pneumonia	*H. alvei*	Penicillin	Cure
Yap 2010 (25)	73 F	Diabetic nephropathy	Fever, abdominal pain, and turbid dialysate	Peritoneal dialysis-related peritonitis	*H. alvei*	Intraperitoneal ceftazidime and amikacin	Cure
Al Saad 2024 (26)	41 F	NA	Recurrent left breast painful swelling, pouring yellowish discharge	Granulomatous mastitis	Streptococcus pyogenes+*H. alvei*	Ampicillin and clavulanic acid + Ciprofloxacin	Cure
Our case	27 F	Gestational diabetes mellitus	Fever, lower abdominal pain	Acute chorioamnionitis, sepsis shock	*H. alvei*, *E. coli* (mixed infection)	Meropenem+piperacillin/tazobactam 17 days total duration	Cure

M = male, F = female, NR = not reported.

This case demonstrates the interaction between host susceptibility and the virulence of opportunistic pathogens in high-risk obstetric environments. Studies indicate that GDM impairs innate immune function, while hyperglycemia reduces neutrophil chemotaxis and phagocytic activity, thereby weakening host defense capabilities. Additionally, GDM is associated with vaginal microbiota dysbiosis, characterized by decreased protective lactobacilli and elevated pH levels, which diminishes colonization resistance and promotes overgrowth of opportunistic pathogens such as *H. alvei* (11).

*H. alvei* carries multiple virulence factors and typically remains dormant in symbiotic states, but becomes pathogenic when the mucosal barrier is compromised, such as premature rupture of membranes. The bacterium harbors genes encoding Vero cell toxins, flagellar motility-associated proteins, and macromolecular secretion systems (including type III and type VI), which collectively mediate its adhesion, invasion, and toxin-induced tissue damage capabilities (12).

Clinically, *H. alvei* infection can rapidly progress to severe sepsis and multiple organ dysfunction, with cases reported in immunocompromised patients and neonates (1, 13–15). This case highlights the need for heightened vigilance when detecting this bacterium in high-risk obstetric patients, as it can quickly transition from a colonizing bacterium to an invasive pathogen, necessitating prompt antimicrobial therapy.

The empirical antibiotic selection for obstetric sepsis should balance broad-spectrum coverage with rational drug use principles. In this case, meropenem was chosen for treatment due to the patient’s history of vaginal colonization with *H. alvei*, high risk of multidrug resistance, and rapid progression to septic shock.

Carbapenems serve as first-line therapy for severe sepsis of unknown etiology during pregnancy, particularly in cases suspected of Gram-negative bacterial infection. They exhibit reliable antibacterial activity against Enterobacteriaceae. Based on the antimicrobial susceptibility results, the subsequent step-down regimen was adjusted to piperacillin/tazobactam, which maintained efficacy against *H. alvei* and *E. coli* while reducing carbapenem exposure. This regimen adheres to the principles of antimicrobial stewardship: early broad-spectrum therapy followed by targeted step-down therapy to minimize the risk of drug resistance.

In addition to *H. alvei*, the potential pathogenic role of *E. coli* co-infection in this case warrants further elaboration. *E. coli* is a well-established pathogen responsible for chorioamnionitis and maternal sepsis, particularly in the setting of preterm premature rupture of membranes (14–16). It possesses potent virulence factors, including lipopolysaccharide, which can trigger a robust systemic inflammatory response and lead to septic shock. In the present case, the patient’s rapid hemodynamic deterioration (requiring norepinephrine), extreme elevation of procalcitonin, and development of paralytic ileus can be adequately explained by the presence of *E. coli* alone.

Notably, the patient had a history of vaginal dysbiosis with overgrowth of *H. alvei* prior to delivery, which may have facilitated subsequent *E. coli* invasion following membrane rupture. We acknowledge that direct evidence for synergistic effects between *H. alvei* and *E. coli* is lacking in this single case report. Therefore, a more conservative interpretation is that this represents a polymicrobial intrauterine infection in which *E. coli* likely played a major pathogenic role, whereas the distinct contribution of *H. alvei* remains uncertain. This highlights the importance of comprehensive microbiological diagnosis and consideration of all isolated pathogens when managing obstetric sepsis.

A systematic review of 14 previously documented cases of extraintestinal *H. alvei* infection reveals a spectrum of clinical presentations, including pneumonia (4 cases), sepsis/bacteremia (4 cases), and intra-abdominal infections such as cholecystitis, abscess, and peritonitis (4 cases). Less common presentations include endocarditis, urinary tract infection, and granulomatous mastitis. Most cases occurred in immunocompromised individuals or those with underlying comorbidities, including diabetes mellitus, chronic kidney disease, malignancy, and HIV infection. Advanced age and the presence of indwelling medical devices (e.g., pacemakers, peritoneal dialysis catheters) were also significant risk factors.

A comparison of this case with the 14 previously reported extraintestinal *H. alvei* infections reveals several distinctive features. First, this is the first documented case of *H. alvei* chorioamnionitis with septic shock, expanding the known clinical spectrum of this pathogen to include obstetric infections. Second, while polymicrobial infection occurred in several prior cases (e.g., with *Enterococcus faecalis*, Klebsiella, or SARS-CoV-2), the combination of *H. alvei* and *E. coli* in the obstetric setting is unique. Third, despite the severity of presentation—septic shock, paralytic ileus, and multi-organ dysfunction—the patient achieved favorable outcomes with timely intervention, contrasting with three fatal cases that all involved elderly patients with advanced underlying diseases, including pancreatic cancer, chronic obstructive pulmonary disease, and coronary artery disease.

Analysis of fatal cases revealed that major risk factors for poor prognosis included: age ≥70 years, concurrent malignant tumors, chronic organ dysfunction, and delayed infection source control. In contrast, younger patients without comorbidities often achieved recovery even with severe manifestations such as septic shock when promptly treated with antibiotics and supportive care. Literature reports indicate effective antibiotics include piperacillin/tazobactam, carbapenems, fluoroquinolones, and cephalosporins. This case demonstrated favorable outcomes after early use of meropenem followed by step-down therapy to piperacillin/tazobactam, confirming the importance of timely intervention and the patient’s baseline condition.

## 4 Limitations

Several limitations of this study should be acknowledged. First, identification of *H. alvei* was based on conventional culture and biochemical methods without molecular confirmation (e.g., 16S rRNA gene sequencing, MALDI-TOF mass spectrometry, or whole-genome sequencing). Given the phenotypic similarity of *H. alvei* to other Enterobacteriaceae, misidentification cannot be entirely excluded. Second, as a single case report, the findings cannot be generalized to all pregnant patients with *H. alvei* colonization. Third, the polymicrobial nature of the infection (co-infection with *E. coli*) precludes definitive attribution of specific clinical features to *H. alvei* alone, and direct evidence for synergistic effects is lacking. Fourth, no quantitative microbiological data or strain typing was performed to confirm whether the same *H. alvei* strain was present in antenatal vaginal swab and postnatal blood/placental cultures. Finally, the retrospective design is subject to potential recall and documentation bias.

## 5 Conclusion

This case highlights the diagnostic and therapeutic challenges of obstetric sepsis caused by rare opportunistic pathogens, such as *H. alvei*. The study demonstrates the necessity of a multidisciplinary collaborative strategy, including early infection control, broad-spectrum antibiotic use, and close monitoring of septic shock and paralytic ileus. The research not only expands our understanding of *H. alvei* infection but also reveals its potential to cause severe intrauterine infections in susceptible populations. The findings underscore the importance of heightened vigilance and timely intervention in high-risk obstetric cases.

## Data Availability

The original contributions presented in the study are included in the article/Supplementary material, further inquiries can be directed to the corresponding authors.
