# Supplementary figures and images for: Hafnia alvei infection complicated by chorioamnionitis and septicemia: a case report and review of literature
Source: Front Med (Lausanne). 2026 Jun 8;13:1840743. doi: 10.3389/fmed.2026.1840743 (PMC13284127; doi:10.3389/fmed.2026.1840743)

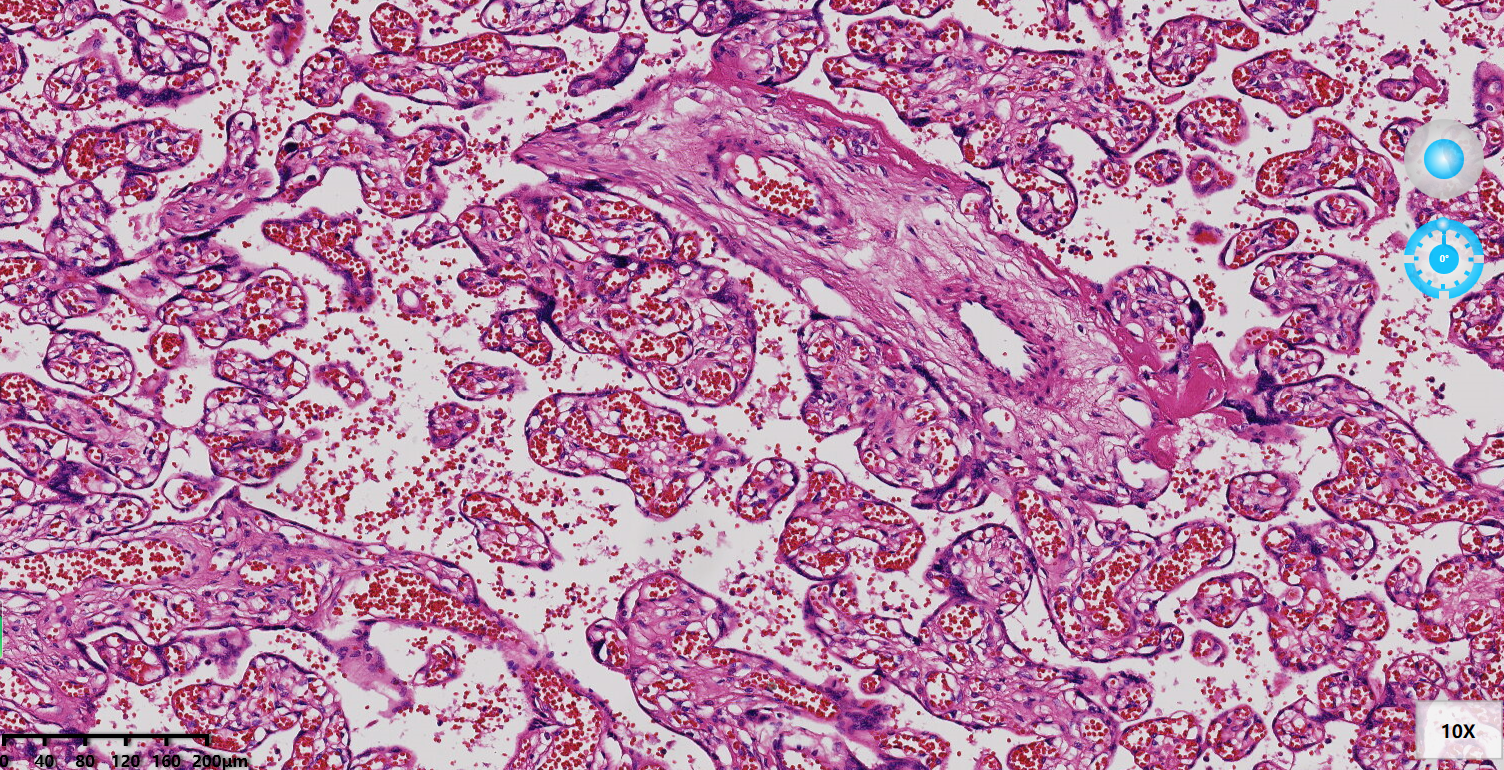

Supplement: Supplementary file 1 [file Data_Sheet_1.ZIP › 新建文件夹/捕获A 10X.PNG]

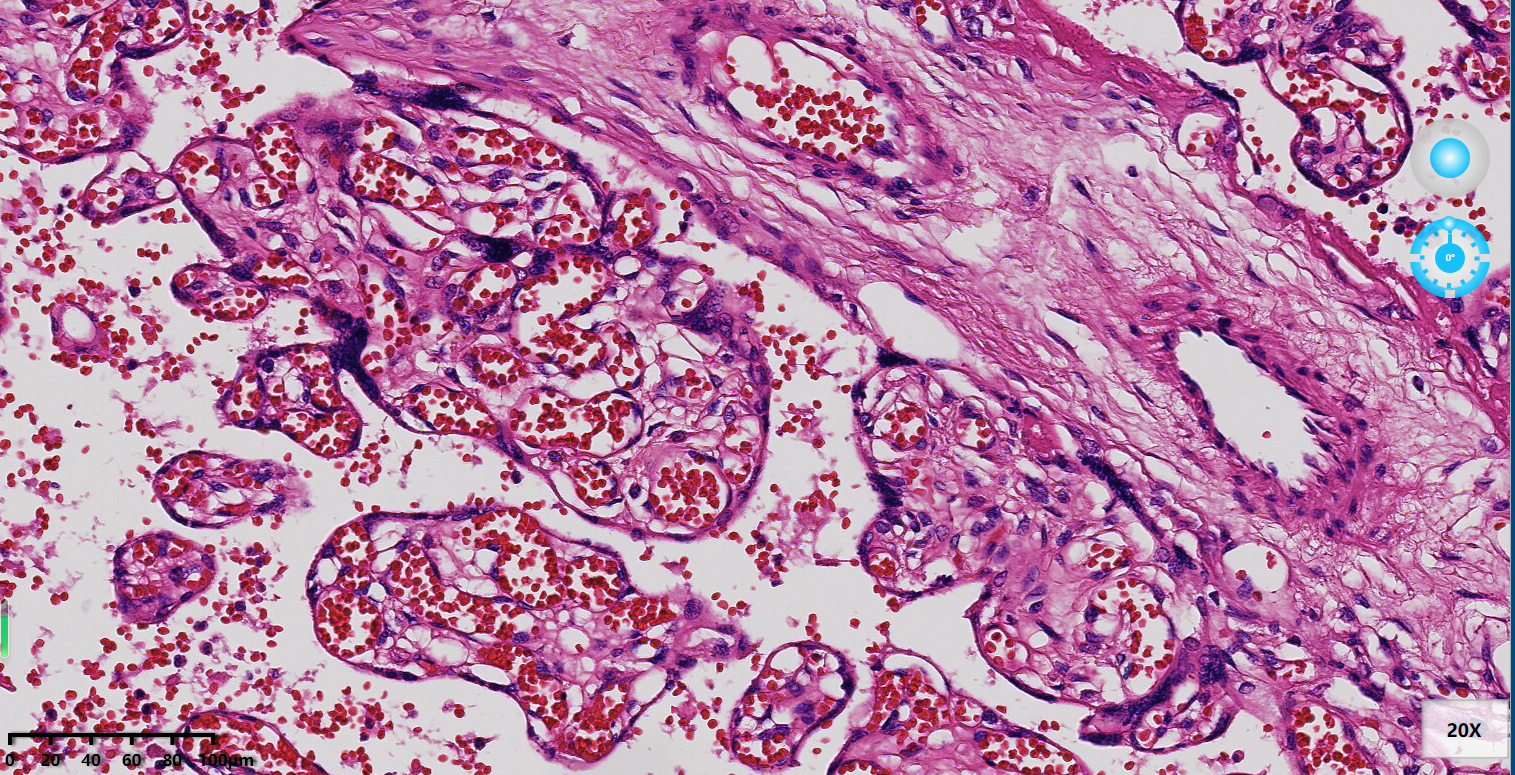

Supplement: Supplementary file 1 [file Data_Sheet_1.ZIP › 新建文件夹/捕获A 20X.PNG]

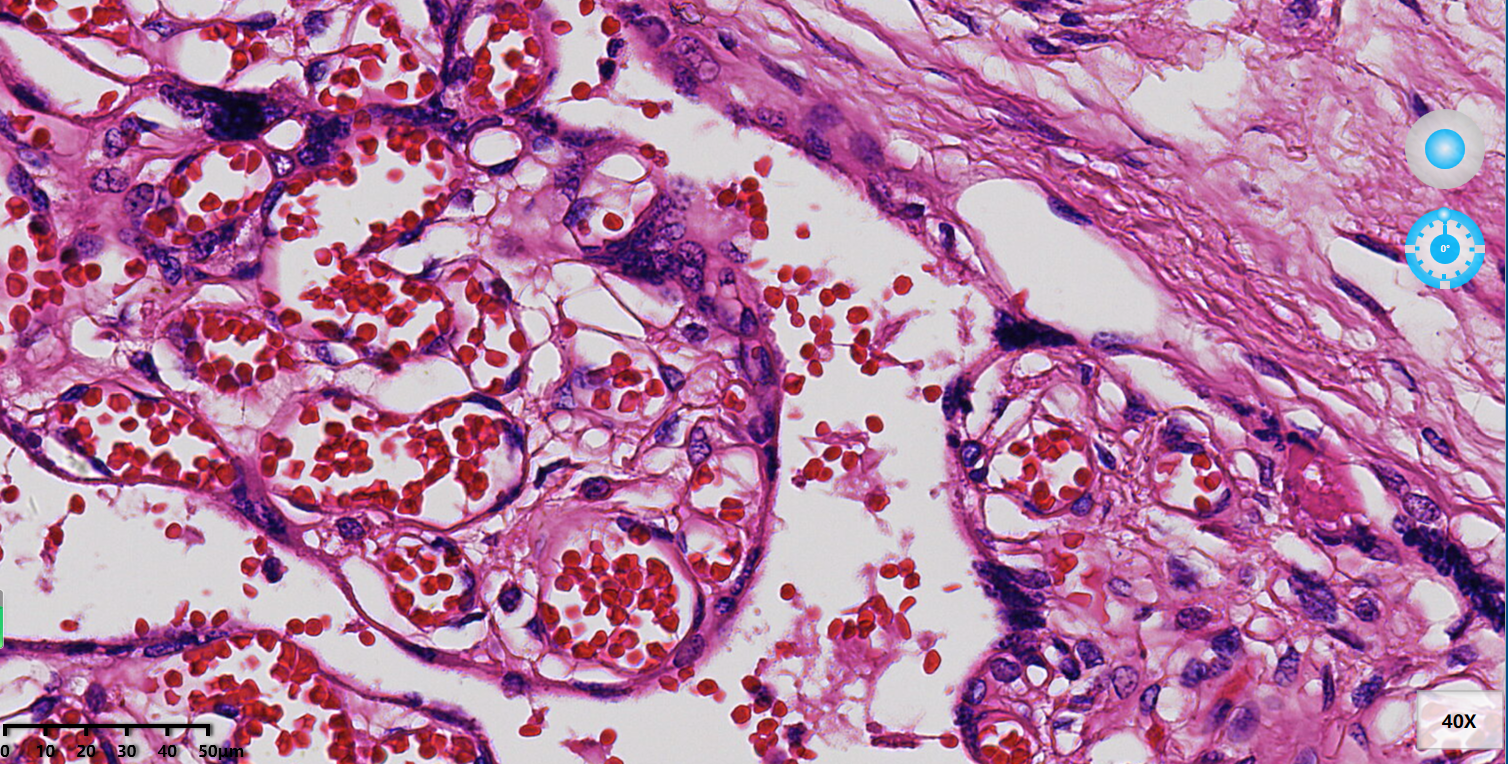

Supplement: Supplementary file 1 [file Data_Sheet_1.ZIP › 新建文件夹/捕获A 40X.PNG]

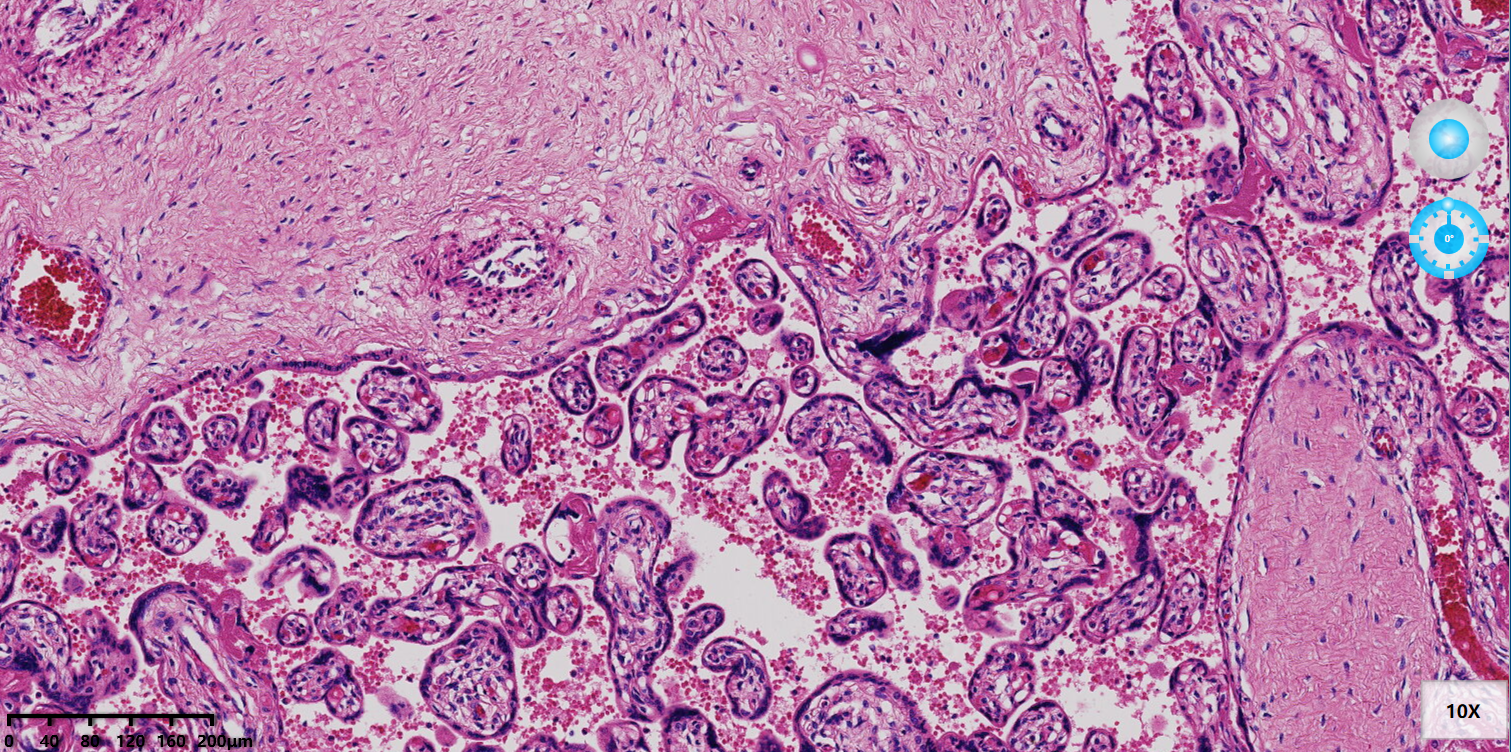

Supplement: Supplementary file 1 [file Data_Sheet_1.ZIP › 新建文件夹/捕获B 10X.PNG]

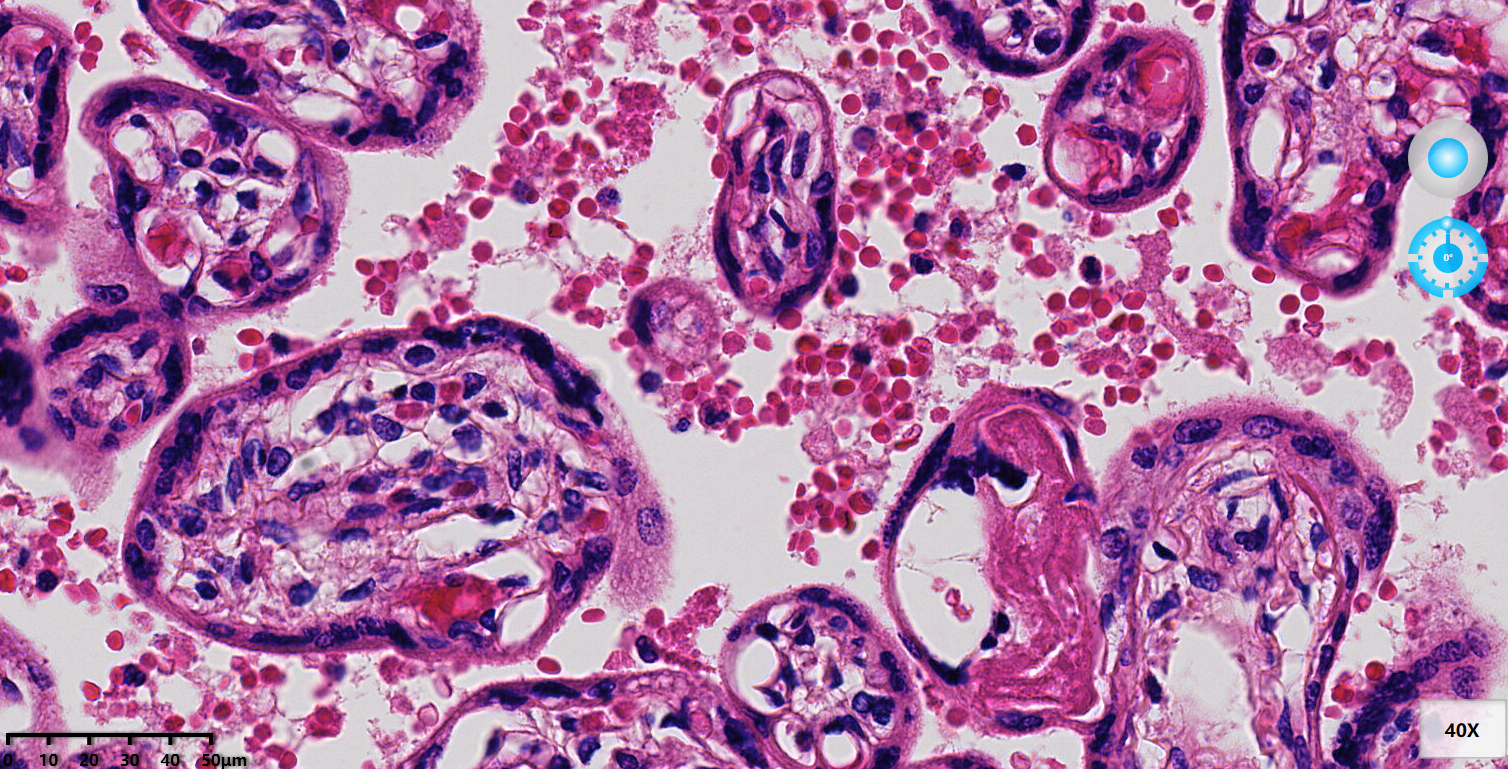

Supplement: Supplementary file 1 [file Data_Sheet_1.ZIP › 新建文件夹/捕获B 40X.PNG]

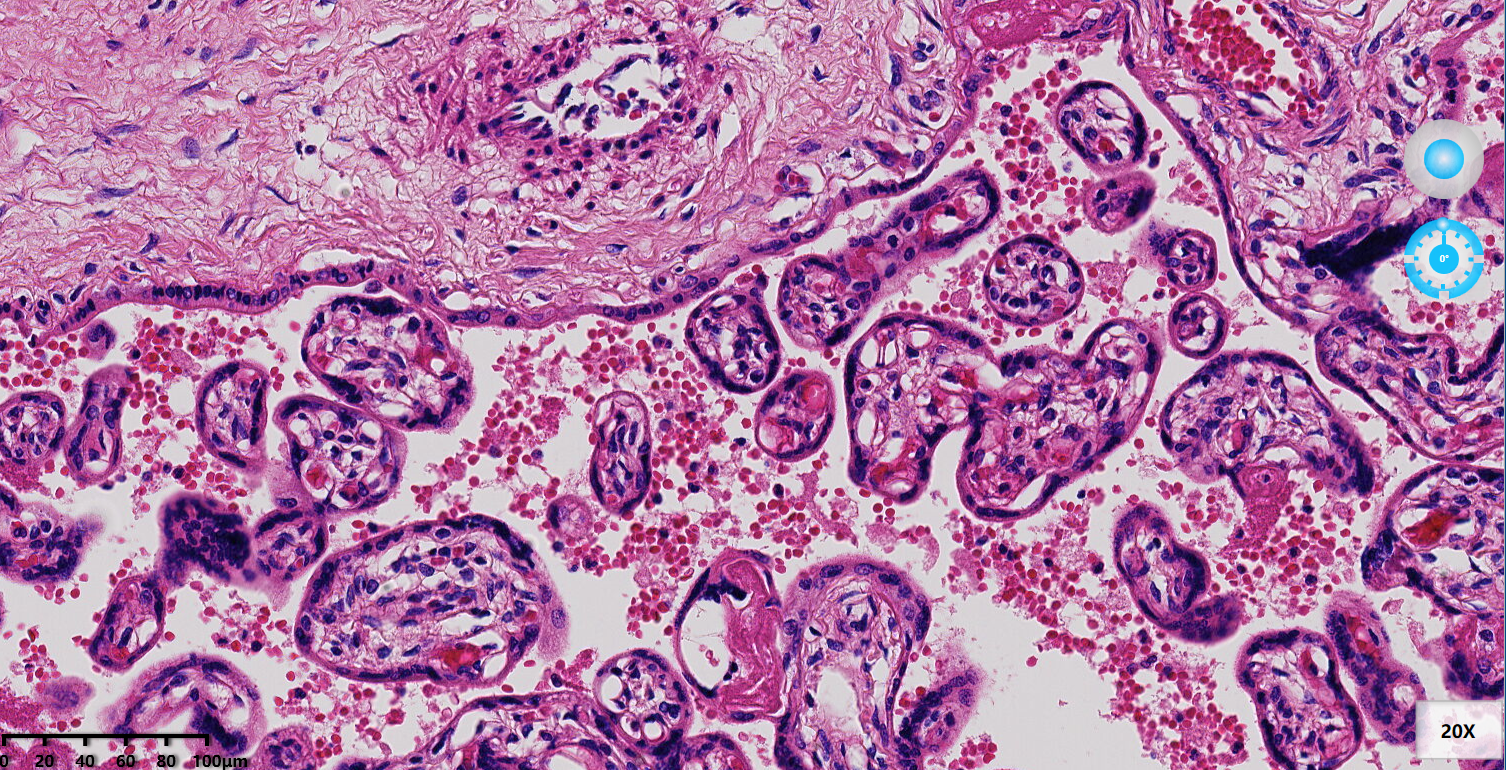

Supplement: Supplementary file 1 [file Data_Sheet_1.ZIP › 新建文件夹/捕获B20X.PNG]
